# Physical activity and academic achievement: an analysis of potential student- and school-level moderators

**DOI:** 10.1186/s12966-022-01348-3

**Published:** 2022-08-30

**Authors:** Hannah K. Behringer, Emilie R. Saksvig, Peter J. Boedeker, Paul N. Elish, Christi M. Kay, Hannah G. Calvert, Adria M. Meyer, Julie A. Gazmararian

**Affiliations:** 1 Department of Epidemiology, Rollins School of Public Health, Emory University, 1518 Clifton Road, Atlanta, GA 30322 USA; 2 Department of Curriculum, Instruction, and Foundational Studies, College of Education, Boise State University, Boise, ID USA; 3 HealthMPowers, Norcross, GA USA; 4 Center for School and Community Partnerships, College of Education, Boise State University, Boise, ID USA

**Keywords:** Physical activity, Academic achievement, Elementary students, Grades, Accelerometers, Socioeconomic status

## Abstract

**Background:**

Many children do not engage in sufficient physical activity, and schools provide a unique venue for children to reach their recommended 60 daily minutes of moderate-to-vigorous physical activity (MVPA). Prior research examining effects of MVPA on academic achievement is inconclusive, and few studies have investigated potential moderators of this relationship. This study examined whether student-level characteristics (gender, race/ethnicity, free/reduced-price lunch status) and school-level characteristics (proportion of students qualifying for free/reduced-price lunch, physical activity environment and opportunities) moderate the relationship between MVPA and academic achievement.

**Methods:**

In a large, diverse metropolitan public school district in Georgia, 4,936 students in Grade 4 were recruited from 40 elementary schools. Students wore accelerometers to measure school-day MVPA for a total of 15 days across three semesters (fall 2018, spring 2019, fall 2019). Academic achievement data, including course marks (grades) for math, reading, spelling, and standardized test scores in writing, math, reading, and Lexile (reading assessment), were collected at baseline (Grade 3, ages 8–9) and at follow-up in Grade 4 (ages 9–10). Standardized test scores were not measured in Grade 5 (ages 10–11) due to COVID-19-related disruptions. Multilevel modeling assessed whether student-level and/or school-level characteristics were moderators in the cross-sectional and longitudinal MVPA-academic achievement relationship.

**Results:**

Cross sectional analyses indicated that the MVPA and AA relationship was moderated only by student Hispanic ethnicity for Grade 4 fall spelling marks (β = -0.159 *p* < 0.001). The relationship for Grade 4 fall spelling marks was also moderated by school physical activity opportunities (β = -0.128 (*p* < 0.001). Longitudinally, there was no significant moderation of the MVPA-academic achievement. A relationship by student gender, free/reduced-price lunch status, race/ethnicity; nor for school-level factors including proportion of students qualifying for free/reduced-price lunch, physical activity environment, and physical activity opportunities.

**Conclusions:**

Overall, our results did not suggest that student- or school-level characteristics moderate the MVPA-academic achievement relationship. While statistically significant results were observed for certain outcomes, practical differences were negligible. In this population, school-based MVPA does not appear to differently affect academic performance based on student gender, race/ethnicity, free/reduced-price lunch, nor school characteristics.

**Trial registration:**

This study was registered with the National Institutes of Health (NIH) ClinicalTrials.gov system, with ID NCT03765047. Registered 05 December 2018—Retrospectively registered.

## Background

Regular physical activity during childhood is associated with positive health outcomes including but not limited to strong bones and muscles, cardiometabolic health, healthier weight, increased cardiorespiratory fitness, and reduced risk of depression [1]. Physical activity habits during childhood may also have implications for health and activity levels during adulthood [2–5], further highlighting the importance of promoting active lifestyles early in life.

Despite the well-known benefits of being active, most US children do not meet physical activity guidelines of 60 min of MVPA per day [6]. As elementary-aged children in America spend about seven hours per day in a school environment, there is great potential to promote healthy behaviors and provide student physical activity opportunities in this setting [7, 8]. With pressures to meet academic standards, many schools aim to increase academic instructional time, often at the expense of physical education and recess time [1, 9]. However, there is no evidence suggesting that physical education time has adverse academic or cognitive effects [10]. Evidence of a positive association between school-based MVPA and academic achievement in addition to the well-cited health benefits may encourage school administrators to adjust policies for further promotion of MVPA in schools.

Research examining the effects of engaging in MVPA over time has not demonstrated consistent effects on academic outcomes. Reviews and meta-analyses from the last decade have reported small, positive relationships between long-term physical activity engagement and academic achievement [11–19]. Other studies have been unable to draw definitive conclusions or did not observe any significant relationship between physical activity and academic achievement [20–24]. Inconsistencies in the literature may be a result of limitations in sample size [11, 22, 23], quality of study designs and methodology [14, 22, 24], subjective measurement of physical activity [13, 14, 20], unspecified physical versus light physical activity or MVPA [14], inadequate follow-up times [11, 22, 23], and inconsistent academic achievement measures [17, 20, 25]. In addition, few studies have closely examined the roles of covariates such as socioeconomic status (SES), sex, and type of physical activity.

Despite the extensive yet inconclusive body of literature surrounding the complex physical activity-academic achievement relationship, potential moderators of this association have rarely been considered. Results from such analyses could inform targeted physical activity interventions to benefit certain subgroups [22]. For instance, examining gender as a moderator is of interest given findings that suggest that an active learning environment may benefit boys more than girls [26]. It has been hypothesized that the traditional, inactive learning environment of schools may be better suited to girls [27, 28], potentially due to gendered responses to the classroom setting. Activity levels tend to be higher among boys [29], which may provide an academic disadvantage to attentiveness in a traditional classroom setting and an advantage in a more active setting. Results of a 2018 randomized controlled trial evaluating the effects of a school-based physical activity program aligned with these hypotheses, indicating a higher increase in academic performance for boys compared to girls [30]. However, a different study found that the relationship between participation in a curriculum-based physical activity intervention and passing standardized tests was only significant among girls, suggesting that girls may in fact reap greater academic benefits than boys from increased physical activity [31]. Notably, these studies have evaluated moderation in the context of specific interventions targeted at active learning rather than physical activity levels in general. There is still much to learn about the role of gender in the physical activity-academic achievement relationship.

In addition, few studies have examined SES and race/ethnicity as moderators of the physical activity-academic achievement relationship. Low-income and minority children tend to have both lower physical activity levels and worse academic outcomes compared to high-SES, white children [32–34]. Assessing how physical activity levels may differently impact academic achievement of low SES and minority racial/ethnic groups can inform steps to close the achievement gap [35]. One study found that effects of fitness on academics were stronger among lower-income students than among students in the higher income group [36]. However, another study indicated that physical fitness test results were positively related to academic achievement, regardless of student SES [37].

School level characteristics such as SES and environment may also be considered as potential moderators of the MVPA-academic achievement relationship. The Whole School Whole Community Whole Child Model emphasizes a collaborative approach between schools and communities to promote both student health and learning. It outlines how academic achievement is dependent on many factors, including quality learning, physical education and physical activity, and community environments, among others [38]. Students in lower SES schools may not have access to as many of these resources, potentially resulting in their academic achievement having a different sensitivity to MVPA compared to that of their higher-SES counterparts. Findings from one study indicated that school-level academic achievement indeed may be affected more by cardiorespiratory fitness in high-SES schools than in low-SES schools but found no differences by school racial composition [39]. However, these studies have focused on fitness, a measure distinct from physical activity. One examination reported a positive relationship between MVPA and academics among minority and low-income children, suggesting that promoting physical activity in schools that mostly serve these groups of children may benefit both student health and academics [40]. However, more work is needed to elucidate the role of school-level moderators to assess if and how physical activity levels may have different effects in different school environments.

This study extends a previous analysis examining the relationship between MVPA and academic outcomes in a diverse population of 4,936 students enrolled in Grade 4 (ages 9–10) fall and followed until Grade 5 (ages 10–11) fall [41]. In this study, we examine whether student-level characteristics (gender, race/ethnicity, and SES) and/or school-level characteristics (SES via percent of students eligible for free/reduced-price lunch, physical activity environment and opportunities) moderate the relationship between student MVPA and academic outcomes. MVPA, as opposed to all physical activity, was evaluated to align with CDC recommendations of 60 min of daily MVPA for children [42]. This relationship was measured cross-sectionally in Grade 4 for course marks (grades) and standardized test scores, and longitudinally from Grade 4 to Grade 5 in teacher-assigned classroom marks.

## Methods

### Study design

Students from 40 public elementary schools from one school district in Metro Atlanta were prospectively followed from Grade 4 to Grade 5 over an 18-month period. The evidence-based *Health Empowers You!* intervention was implemented in 20 intervention schools with the goal of sustainably increasing student MVPA time. This present study is not an evaluation of the intervention, but an investigation of the MVPA-academic achievement relationship and its potential moderators. Details about school and student recruitment are provided in an earlier manuscript [35]. All 40 schools were included in the present study. This study was approved by the school district administration, district Institutional Review Board (IRB), and Emory University IRB.

### Study participants and recruitment

Enrollment consisted of guardian consent and student assent for participation in the MVPA measurement, and authorization for sharing school district data. Academic information (standardized test scores, reading Lexile, course marks, attendance, and tardiness) was deidentified by the district and linked to accelerometer data via unique student identifiers. Data for course marks was collected for three years: Grade 3, Grade 4, and Grade 5, and standardized test score data were collected in Grade 3 and Grade 4. Of all Grade 4 students, 4,936 (76%) provided consent/assent and were enrolled in the study [35].

#### Data sources

Four data sources were used for this study: (1) school district, demographic, and academic assessment data, (2) student-worn accelerometers, (3) the School Physical Activity Policy Assessment (S-PAPA), and (4) the Observational School Environment Checklist (OSEC).

##### School district data

School district data included student standardized test scores, course grades, demographics (sex and race/ethnicity), free/reduced-price lunch status, English language learner status, student with disabilities status, special education participation, and attendance (including days enrolled, absences, and tardiness).

##### Accelerometers

The study used ActiGraph wGT3X-BT 3-axis accelerometers (ActiGraph LLC, Pensacola, FL), worn on the waist. Students wore their assigned accelerometer at the beginning of the school day and removed it before leaving school. *S-PAPA:* A tool used to assess school policy and environmental variables as related to the quantity and quality of children’s school PA. The aim of this instrument is to provide information about specific district and school policy areas, school practices, and environmental conditions that influence school MVPA [43]. Physical activity policy surveys were completed by a classroom teacher, physical education teacher, and the school’s principal during spring of Year 1 and Year 2 of the study.

##### OSEC

An observational audit tool used to assess schools’ environments for physical activity [44]. In-person facility observations were done during spring of Year 1 and Year 2 of the study by physical activity specialists.

#### Measures

##### Exposure

Our exposure was accelerometer measured MVPA. Criteria for a valid day required students to have worn the accelerometer for at least 80% of the school day. Students needed at least 3 valid days of wear time during the 5-day measurement period each semester to be included in analyses for that semester. A single measure of mean MVPA minutes were calculated in each semester (fall 2018, spring 2019, and fall 2019) for students who met the 3-day criteria. Students wore their assigned accelerometer for 15 days across all three semesters. ActiLife software was used to download and score the data and filtered such that only school-day minutes were used in scoring. As in previous studies, data were collected in 15-s epochs and scored using Evenson cut points for activity thresholds [45, 46]. Additional measurement and coding details can be found in previous manuscripts [35, 41].

##### Primary outcomes

Two primary outcomes were examined; Grade 4 and 5 course marks and Grade 4 standardized test scores. Georgia Milestones tests were not administered in spring 2020 due to the COVID-19 pandemic, so Grade 5 standardized test and Lexile (reading) scores were not available. Course marks were assigned by teachers each semester for math, reading, spelling, and writing on a 100-point scale. Per district protocol, all teachers used the same percentages for assigning grades and the same grading scale. In addition, the curriculum and professional development were the same for all teachers, helping to reduce bias associated with using teacher grades as an outcome. Further details surrounding academic outcomes can be found in the main effects paper [41].

##### Moderators

Potential student-level moderators of the relationship between MVPA and academic achievement included gender, free/reduced-price lunch status as a proxy for SES, and race/ethnicity. Students were eligible for free/reduced-price lunch if their family household income was at or below 185% of the federal poverty level. Gender was documented as either “male” or “female”, free/reduced-price lunch status was dichotomized as “yes”, or “no”. Race and ethnicity were classified as “Asian”, “Black”, “Hispanic”, “Mixed”, or “White”.

School-level moderators evaluated included proportion of students qualifying for free/reduced-price lunch, school physical activity opportunities, and school physical activity environment. S-PAPA surveys were completed by classroom teachers (six questions), physical education teachers (nine questions), and administrators (five questions) and used to measure physical activity opportunities in the context of physical education and recess. Answers were assigned scores and summed to create physical education and recess scores, with higher scores corresponding to environments more conducive to physical activity. Scores were analyzed in separate models as either continuous or categorical, with the categorical specification based on a median split. The OSEC was used to measure schools’ physical activity environment. This observational audit consisted of 10 questions divided into three sections: atmosphere (eight questions), accessibility (seven questions), and advertisement (three questions). Schools received scores for each of the three categories, with higher scores corresponding to higher-quality environments [44]. Each of these three components was analyzed as a potential moderator.

##### Covariates

Student gender, free/reduced-price lunch status, race/ethnicity, disability status, English language learner status, participation in special education courses, teacher departmentalization, and prior achievement, absenteeism, and tardiness were controlled for in all models. Physical or learning disability and English language learner status were dichotomized as “yes” or “no.” Special education participation was incorporated as a variable ranging from zero to four based on the number of special education courses in which students were enrolled. Some teachers were departmentalized, meaning students rotate between teachers for certain subjects, and departmentalization entered the model as a student characteristic. Prior achievement was defined as the previous year’s (Grade 3) course marks or standardized test score. Finally, a student’s prior absenteeism and tardiness were measured by percent days absent and tardy in Grade 3. School-level covariates included percentage of students who were female, Black, Hispanic, and free/reduced-price lunch-eligible. Analyses also accounted for school intervention or control status.

#### Statistical analysis

Descriptive statistics were first calculated for the sample, and the data examined for missing values. Variables were missing data either because (1) students were not enrolled in the participating schools for the entire study period or (2) their observation did not meet inclusion criteria (e.g., no accelerometer data). Of the 4,936 students, 87.5% (*n* = 4,320) had a valid accelerometer measure in Grade 4 fall, 77.0% (*n* = 3,800) in Grade 4 spring, and 73,7% (*n* = 3,588) in Grade 5 fall. Accelerometer data for Grade 5 fall had the highest missingness of any variable. Multiple imputation addressed missing data. Twenty imputed datasets were created using the multilevel multiple imputation program Blimp [47]. Implausible imputed values were set to variables’ upper or lower bounds. Descriptive statistics are presented for the non-imputed data. Results for analyses conducted using listwise deletion and multiply imputed data were similar; therefore, results from analyses with multiple imputation are presented. Cross-sectional analyses are conducted predicting student achievement within fourth grade with MVPA measured in 4^th^ grade. Longitudinal analyses utilize fourth grade MVPA to predict 5^th^ grade fall achievement.

Given the nested structure of the data with students contained within schools, multilevel models with two-levels were fit. Moderation is assessed by the interaction of student MVPA and the potential moderator. The coefficient of the interaction describes the difference in the observed MVPA-academic achievement association for various levels of the moderator. For student-level moderators, this requires the product of MVPA and the potential student-level moderator. For school-level moderators, cross-level interactions are required wherein the coefficient of MVPA is allowed to vary across schools and its variability be explained by school-level characteristics. To account for multiple tests, statistical significance was determined using a Bonferroni adjusted critical *p*-value of 0.00038.

## Results

### Sample characteristics

Data from 4,936 Grade 4 students from 40 public schools were included in the analyses (Table 1). Gender was evenly balanced (50.0% female), and 12.2% of participants identified as Asian, 25.2% Black, 33.2% Hispanic, 4.3% Mixed, and 24.8% White. About half (53.1%) of the students qualified for free/reduced-price lunch during the study period, about a third (35.1%) were current or former English language learners, and 12.9% had learning or physical disabilities. At the school level, an average of 56.1% of students were eligible for free/reduced-price lunch; and student populations were, on average, 48.6% female, 27.3% Black, and 32.2% Hispanic.

**Table 1:** Student and school demographics, academic achievement, and physical activity data, Grades 4 to 5

**Student-level data, *n* = 4,936 **^**a**^		
**Variable**	**Count / average**	**% / SD**
**Sex**		
Female	2,466	(50.0%)
Male	2,465	(49.9%)
**Race/Ethnicity**		
Asian	601	(12.2%)
Black	1,243	(25.2%)
Hispanic	1,640	(33.2%)
Mixed	213	(4.3%)
White	1,226	(24.8%)
**Free/Reduced-Price Lunch Recipient**		
Yes	2,622	(53.1%)
No	2,309	(46.8%)
**Grade 3 Average Course Marks**		
Math	83.5	(9.4)
Reading	82.9	(9.0)
Spelling	87.9	(9.0)
Writing	84.4	(7.8)
**Grade 4 Fall Course Marks**		
Math	81.5	(12.0)
Reading	80.9	(10.3)
Spelling	86.7	(11.3)
Writing	83.2	(8.9)
**Grade 4 Spring Course Marks**		
Math	83.2	(10.6)
Reading	81.8	(9.9)
Spelling	87.3	(10.2)
Writing	83.8	(8.7)
**Grade 5 Fall Course Marks **^**b**^		
Math	82.0	(11.5)
Reading	82.4	(9.4)
Spelling	87.6	(9.6)
Writing	84.9	(8.2)
**Grade 3 Standardized Test Scores **^**c**^		
Math	541.4	(49.9)
English Language Arts	527.1	(58.2)
Lexile	728.6	(219.6)
**Grade 4 Standardized Test Scores **^**c**^		
Math	549.2	(53.7)
English Language Arts	535.2	(55.5)
Lexile	897.2	(221.1)
**Average daily moderate-to-vigorous physical activity (MVPA) minutes**		
Grade 4 Fall	21.1	(9.2)
Grade 4 Spring	21.9	(10.0)
Grade 5 Fall	18.9	(8.9)

^a^ Not all tabulations add to 4,936 due to missing data

^b^ Due to COVID-related disruptions, Grade 5 Spring course marks were not available

^c^ Due to COVID-related disruptions, no standardized tests were conducted in Grade 5

Average course marks among the sample were similar for Grade 3 fall and spring, Grade 4 fall and spring, and Grade 5 fall. Mean scores were lowest in reading (Grade 3 mean: 82.9, Grade 4 mean: 81.4, Grade 5 fall mean: 82.4) and highest in spelling (Grade 3 mean: 87.9, Grade 4 mean: 87.1, Grade 5 fall mean: 87.6). Students’ respective average scores on Grade 3 and 4 standardized tests were 541.4 and 549.2 for math, 527.1 and 535.2 for English language arts, and 728.6 and 897.2 for Lexile.

A slight decline in average daily MVPA throughout the study period was observed. On average, students engaged in 21.14 min of MVPA in Grade 4 fall, 21.85 min in Grade 4 spring, and 18.91 min in 5^th^ grade fall. Among intervention schools, average daily MVPA was 22.6, 24.1, and 21.5 min for Grade 4 fall, Grade 4 spring, and Grade 5 fall, respectively. Results from our examination of the study’s main effects suggested school-day MVPA does not have an association with course grades nor standardized test scores [41].

### Moderation analyses

At the student level, cross-sectional analyses showed no significant moderation of the MVPA-academic achievement relationship by gender nor FRL status for course marks. The relationship between MVPA and academic achievement was found to be moderated by student Hispanic ethnicity for one of the 15 academic achievement outcomes measured, Grade 4 fall spelling marks (β = -0.159; *p* < 0.0001). Cross sectional analysis showed significant moderation by school physical education policies for Grade 4 fall spelling marks (β = -0.127; *p* < 0.0001). There was no other evidence of moderation by school recess or physical education policies for both Grade 4 and 5 when assessing the academic outcome of course marks (Table 2). No statistically significant moderation by any of the three school environment variables, measured by the OSEC survey, was observed for any of the course mark academic outcomes (data not shown).

**Table 2:** Cross-sectional associations of Grade 4 fall MVPA and course marks, moderated by student and school characteristics ^a^

	**Math**		**Reading**		**Writing**		**Spelling**	
	Association (SE)	*p*	Association (SE)	*p*	Association (SE)	*p*	Association (SE)	*p*
**Student Characteristics**								
MVPA	-0.053 (0.019)	0.005	-0.044 (0.016)	0.008	-0.060 (0.016)	0.000	-0.067 (0.022)	0.002
Gender	-0.524 (0.242)	0.031	0.676 (0.211)	0.001	1.143 (0.208)	0.000	0.560 (0.272)	0.040
MVPA * gender	-0.003 (0.027)	0.920	-0.014 (0.024)	0.545	0.009 (0.024)	0.688	-0.003 (0.032)	0.937
MVPA	-0.024 (0.021)	0.264	-0.039 (0.019)	0.040	-0.051 (0.018)	0.005	-0.023 (0.024)	0.343
Student FRL	-0.673 (0.296)	0.023	-0.480 (0.260)	0.064	-0.791 (0.254)	0.002	-0.852 (0.334)	0.011
MVPA * FRL	-0.055 (0.027)	0.047	-0.019 (0.024)	0.415	-0.009 (0.023)	0.691	-0.081 (0.030)	0.007
MVPA	0.008 (0.029)	0.797	-0.013 (0.025)	0.599	-0.046 (0.024)	0.059	-0.002 (0.032)	0.950
Hispanic	-0.721 (0.404)	0.074	-0.843 (0.353)	0.017	-0.735 (0.345)	0.033	-0.432 (0.453)	0.340
Black	-0.837 (0.376)	0.026	-0.589 (0.324)	0.070	-0.635 (0.318)	0.046	0.261 (0.423)	0.536
Asian	2.511 (0.437)	0.000	1.032 (0.380)	0.007	1.445 (0.373)	0.000	2.237 (0.488)	0.000
Mixed	1.043 (0.610)	0.087	0.878 (0.527)	0.096	1.059 (0.519)	0.041	2.015 (0.689)	0.004
MVPA * Hispanic	-0.105 (0.038)	0.005	-0.042 (0.032)	0.190	-0.023 (0.031)	0.461	-0.159 (0.041)	**0.000**
MVPA * Black	-0.101 (0.039)	0.009	-0.061 (0.032)	0.059	-0.013 (0.031)	0.678	-0.037 (0.041)	0.365
MVPA * Asian	-0.028 (0.048)	0.566	-0.049 (0.043)	0.252	0.012 (0.041)	0.778	-0.043 (0.054)	0.433
MVPA * Mixed	0.078 (0.066)	0.237	-0.014 (0.057)	0.800	-0.002 (0.056)	0.968	0.015 (0.074)	0.844
**School Characteristics**								
MVPA	-0.054 (0.016)	0.001	-0.049 (0.013)	0.000	-0.056 (0.013)	0.000	-0.068 (0.017)	0.000
School % FRL	-0.009 (0.089)	0.916	-0.038 (0.077)	0.620	-0.099 (0.081)	0.221	-0.207 (0.086)	0.016
MVPA * % FRL	-0.001 (0.001)	0.025	-0.001 (< 0.001)	0.155	0.000 (< 0.001)	0.597	-0.002 (0.001)	0.010
MVPA	-0.037 (0.023)	(0.105)	-0.034 (0.019)	0.075	-0.027 (0.019)	0.148	-0.031 (0.025)	0.205
SPAPA Recess	1.119 (1.220)	0.359	0.772 (1.070)	0.471	-0.297 (1.116)	0.790	1.099 (1.158)	0.343
MVPA*SPAPA Recess	-0.03 (0.02)	0.272	-0.029 (0.025)	0.239	-0.053 (0.024)	0.029	-0.068 (0.032)	0.034
MVPA	-0.018 (0.026)	0.498	-0.002 (0.022)	0.947	-0.018 (0.022)	0.409	0.019 (0.029)	0.502
SPAPA PE	0.911 (1.067)	0.394	0.866 (0.925)	0.349	0.556 (0.975)	0.573	-0.766 (1.047)	0.464
MVPA*SPAPA PE	-0.054 (0.031)	0.079	-0.075 (0.026)	0.006	-0.071 (0.026)	0.033	-0.128 (0.033)	**0.000**

^a^ Models adjusted for student sex, race/ethnicity, FRL status, English Language Learner status, student with disabilities status, Grade 3 absences, Grade 3 tardies, special education course enrollment, school percentage female, school percentage black, school percentage Hispanic, school percentage FRL, school cohort (intervention or control), departmentalization, and prior achievement for the specific academic outcome (e.g., when assessing math grade as outcome, used Grade 3 average math grade). The 95% Confidence Interval for the indirect effect was found using the Monte Carlo method. In bold are the indirect effects found to be statistically significant using a Bonferroni corrected alpha of 0.00038

When examining standardized test score outcomes, there was no evidence of moderation by student gender, free/reduced-price lunch status, nor race/ethnicity. In addition, no statistically significant moderation by any of the school-level variables was observed for the standardized test score outcomes (Table 3).

**Table 3:** Cross-sectional associations of Grade 4 MVPA and Grade 4 standardized test scores, moderated by student and school characteristics ^a^

	**Math**		**ELA**		**Lexile**	
	Association (SE)	*p*	Association (SE)	*p*	Association (SE)	*p*
**Student Characteristics**						
MVPA	-0.237 (0.090)	0.009	-0.148(0.094)	0.115	-0.227 (0.416)	0.585
Student female gender	-8.839 (0.981)	0.000	3.250 (1.050)	0.002	9.657 (4.793)	0.044
MVPA * gender	-0.082 (0.114)	0.472	-0.127 (0.132)	0.340	-0.665 (0.598)	0.267
MVPA	-0.216 (0.104)	0.039	-0.139 (0.109)	0.205	0.018 (0.488)	0.970
Student FRL	-3.714 (1.095)	0.001	-1.081 (1.228)	0.380	-5.421 (5.594)	0.333
Interaction	-0.096 (0.114)	0.400	-0.107 (0.126)	0.397	-0.904 (0.570)	0.114
MVPA	-0.139 (0.132)	0.295	-0.007 (0.143)	0.962	0.701 (0.642)	0.276
Hispanic	-5.503 (1.506)	0.000	-0.828 (1.693)	0.625	-2.566 (7.692)	0.739
Black	-5.496 (1.408)	0.000	0.798 (1.568)	0.612	-0.065 (7.108)	0.993
Asian	5.166 (1.606)	0.001	10.300 (1.802)	0.000	43.931 (8.202)	0.000
Mixed	-0.620 (2.257)	0.784	2.227 (2.522)	0.377	-8.799 (11.460)	0.443
MVPA*Hispanic	-0.273 (0.159)	0.087	-0.320 (0.178)	0.073	-2.150 (0.805)	0.008
MVPA*Black	-0.059 (0.153)	0.700	-0.293 (0.178)	0.100	-1.809 (0.781)	0.021
MVPA*Asian	0.001 (0.192)	0.995	0.134 (0.218)	0.540	0.642 (0.976)	0.511
MVPA*Mixed	-0.507 (0.290)	0.082	-0.399 (0.322)	0.217	-1.116 (1.462)	0.446
**School Characteristics**						
MVPA	-0.269 (0.075)	0.001	-0.197 (0.077)	0.011	-0.480 (0.345)	0.165
School % FRL	-0.064 (0.165)	0.699	-0.055 (0.177)	0.755	-0.349 (0.660)	0.598
MVPA * % FRL	-0.003 (0.002)	0.269	-0.004 (0.003)	0.098	-0.036 (0.012)	0.002
MVPA	-0.561 (2.273)	0.805	0.639 (2.471)	0.796	-1.645 (9.087)	0.856
SPAPA Recess	-0.257 (0.092)	0.006	-0.055 (0.177)	0.202	-0.382 (0.475)	0.422
MVPA*SPAPA Recess	-0.020 (0.869)	0.869	-0.118 (0.135)	0.381	-0.207 (0.599)	0.730
MVPA	-1.045 (2.007)	0.603	-0.959 (2.178)	0.660	-3.727 (7.986)	0.641
SPAPA PE	-0.232 (0.104)	0.026	-0.245 (0.118)	0.039	-0.921 (0.543)	0.091
MVPA*SPAPA PE	-0.020 (0.869)	0.661	-0.068 (0.136)	0.616	0.654 (0.634)	0.303

^a^ Models adjusted for student sex, race/ethnicity, FRL status, English Language Learner status, student with disabilities status, Grade 3 absences, Grade 3 tardies, special education course enrollment, school percentage female, school percentage black, school percentage Hispanic, school percentage FRL, school cohort (intervention or control), departmentalization, and prior achievement for the specific academic outcome (e.g., when assessing math grade as outcome, used Grade 3 average math grade). The 95% Confidence Interval for the indirect effect was found using the Monte Carlo method. In bold are the indirect effects found to be statistically significant using a Bonferroni corrected alpha of 0.00038

Longitudinal analyses indicated that there was no significant moderation of the MVPA-academic achievement relationship by student gender, free/reduced-price lunch eligibility, nor race/ethnicity for any of the academic outcomes. Potential school-level moderators were also not found to be significant in the longitudinal analysis (data not shown).

## Discussion

Results from this study indicate that the relationship between school-day MVPA and academic outcomes does not differ significantly by student- or school-level characteristics. These results from a large, diverse sample contribute valuable information as knowledge of moderation in the MVPA-academic achievement relationship is currently sparse.

Although a few academic outcomes were found to be statistically moderated by student or school characteristics, the magnitudes of the effects were too small to have meaningful, real-world implications. For instance, the coefficient corresponding to school physical education policies for Grade 4 fall spelling marks was -0.128. While this suggests that students in schools with better S-PAPA physical education scores saw larger decreases in spelling marks compared to students in schools with lower S-PAPA scores, this difference translates to only 1.28 points for every 10-min increase in daily MVPA. A 10-min increase in MVPA would be substantial—an increase of about 300% from current levels. Notably, there was little to no evidence of moderation by student gender, eligibility for free/reduced-price lunch, race/ethnicity or school-level factors including physical activity environment and SES in the longitudinal analyses, further indicating that these factors may not moderate this relationship.

Results from previous examinations of moderation of this relationship by student gender are inconsistent. Our results of no moderation by gender conflict with previous research suggesting boys benefitted academically from a school-based activity program more than girls [30], or that MVPA interventions may be particularly beneficial among girls instead [31]. However, both studies used only standardized testing as an academic outcome and evaluated specific interventions rather than the general MVPA-academic achievement relationship. One cross-sectional study also observed that among 9^th^ grade students, academic achievement was associated with vigorous MVPA for girls but not for boys [48]. Paired with our results, this may suggest that moderation by gender might not emerge until adolescence, but more work is needed to clarify this.

Evidence of SES as a moderator of this relationship is also limited. While some previous work has examined the role of SES in the relationship between fitness and academic outcomes, with contrasting results [36, 37, 39], there is a lack of information surrounding SES and the MVPA-academic achievement relationship. Our findings that MVPA levels were not associated with academic outcomes, regardless of SES, align with one review reporting that SES and ethnicity did not have any effect on the MVPA-AA relationship [40]. While this study found an overall positive relationship between MVPA and AA and our results showed no significant relationship [41], it is notable that results were similar across sociodemographic characteristics.

While lacking for MVPA, hypotheses exist for how SES may moderate the fitness-academic achievement relationship in both directions. It has been suggested that higher-SES students are better equipped to balance potentially negative academic effects of low cardiorespiratory fitness due to having more resources at home, therefore being less sensitive to effects of fitness [49]. Conversely, the Whole School, Whole Community, Whole Child model could indicate this relationship is stronger in schools with higher SES. In the context of this framework, it is suggested that students in lower-SES schools may have fewer resources included in this model, and academic benefits that could result from increased fitness may not materialize without important complementary resources. As a result, schools with lower SES may be less likely to see academic effects of fitness changes [39]. It should be emphasized that these hypotheses, while potentially related, focus on student cardiorespiratory fitness. Regular MVPA can increase cardiorespiratory fitness [50], but these are distinct exposures that merit individual assessment. In addition, no previous studies have examined the role of school physical activity environments to our knowledge, but the present results do not provide evidence that these school factors moderate this relationship. While our study suggests promising implications for consistent relationships between MVPA and academic achievement, future work is needed to confirm the role of SES and the school environment.

Our results are encouraging, as they suggest that in-school MVPA may not be meaningfully associated with negative academic effects, regardless of student and school characteristics. Across schools and populations in this diverse, suburban county, school-day physical activity levels may enhance the physical and mental health of students, which has been particularly threatened during the COVID-19 pandemic [51], without detracting from academic progress.

### Strengths

This is the largest study examining the association between accelerometer measured MVPA and academic achievement in the US to date, with a diverse sample of almost 5,000 elementary students: consisting of about a third Black, a third White, and a third Hispanic students. In addition, half of the sampled students qualified for free/reduced-price lunch. Previous studies have tended to report more homogeneous samples of fewer than 700 individuals. Second, much of the previous research has been limited to cross-sectional analyses, and the longitudinal aspect of our study further strengthens the evidence base. Third, utilization of accelerometers to measure MVPA and repeating these measurements across three semesters increases the validity of MVPA measurement. Finally, this study contained a high participation rate at 76% across all Grade 4 students at baseline, limiting selection bias.

### Limitations

A limitation of the present research is that the COVID-19 pandemic shortened the original four-semester intervention to three semesters, limiting the duration of our longitudinal follow-up and preventing administration of 5^th^ grade standardized testing. Second, we only used total average time in MVPA, but it has been suggested that frequency, duration, and intensity of MVPA might influence its relationship with academic achievement [40]. In was not feasible to discretely analyze data for each of these factors due to the large sample size. Future studies could more closely examine the most effective type and dose of MVPA for academic benefits. Third, the 3–5 days of accelerometer data collection per semester may be less accurate for extrapolation to the entire semester than a longer accelerometer measurement period on the student level [52]. However, school day MVPA-tends to be less variable than MVPA data from the full day [17], and the measures were repeated over three semesters. Lastly, MVPA data was only collected during the school day, limiting our ability to draw conclusions about the association of total activity levels with academic achievement. An additional analysis on a small subsample of students whose activity was measured for 24 h indicated that average out of school MVPA was 28 min, thus, differences in leisure time MVPA may have influenced the present results. For instance, associations with academic achievement may not have been detected among children who acquire most of their MVPA outside of school. Therefore, the present findings only inform how MVPA acquired during the school day may relate to academic achievement.

## Conclusion

The relationship between school-day MVPA and academic performance does not differ significantly by student- or school-level characteristics. Schools may be able to protect and promote school time dedicated to MVPA to enhance the well-being of students without meaningfully compromising academic performance, regardless of gender, SES, race/ethnicity, or school characteristics. As we continue to observe low compliance to daily MVPA guidelines among children and the associated negative effects on physical and mental health, it is crucial to leverage the school setting to boost MVPA levels. Enhancing our understanding of the relationship between MVPA and academic outcomes can inform steps to do so.

## Data Availability

Data used in the current study are available and may be obtained from the corresponding author upon reasonable request.

## Abbreviations

CDC: The Centers for Disease Control and Prevention
IRB: International Review Board
MVPA: Moderate-to-vigorous physical activity
OSEC: Observational School Environment Checklist
SES: Socioeconomic status
S-PAPA: School Physical Activity Policy Assessment
